# Linker Histones: The Multiple Binding Modes of the Enigmatic 5th Histone

**DOI:** 10.3390/biom16081181

**Published:** 2026-08-13

**Authors:** Nicholas R. Rugelis, Ashok Kumar, Jeffrey J. Hayes

**Affiliations:** Department of Biochemistry and Biophysics, University of Rochester Medical Center, Rochester, NY 14642, USA; nicholas_rugelis@urmc.rochester.edu (N.R.R.); ashok_kumar1@urmc.rochester.edu (A.K.)

**Keywords:** nucleosome, chromatin, linker histones (H1s), nucleosome core particle, chromatosome, on-dyad binding, off-dyad binding

## Abstract

Chromatin structure is dynamic and regulated by many factors, including enzymes that chemically modify histones and DNA, chromatin remodeling complexes that physically manipulate nucleosomes and chromatin, and non-enzymatic proteins that bind to DNA or nucleosomes to create specialized regions in chromatin. This multifactorial regulation stems from the need for fine-tuned control, which is key in processes including DNA repair, replication, and gene expression. Linker histones are a family of proteins structurally distinct from the core histones that provide a poorly understood layer of regulation in chromatin. In this review, we introduce the basics of chromatin structure, what is known about how linker histones (H1s) bind to nucleosomes and influence chromatin, and how the individual domains within H1s contribute to these activities. We especially focus on recent studies describing canonical and alternative H1-nucleosome binding and their potential roles in chromatin.

## 1. Introduction

**Figure 1:**
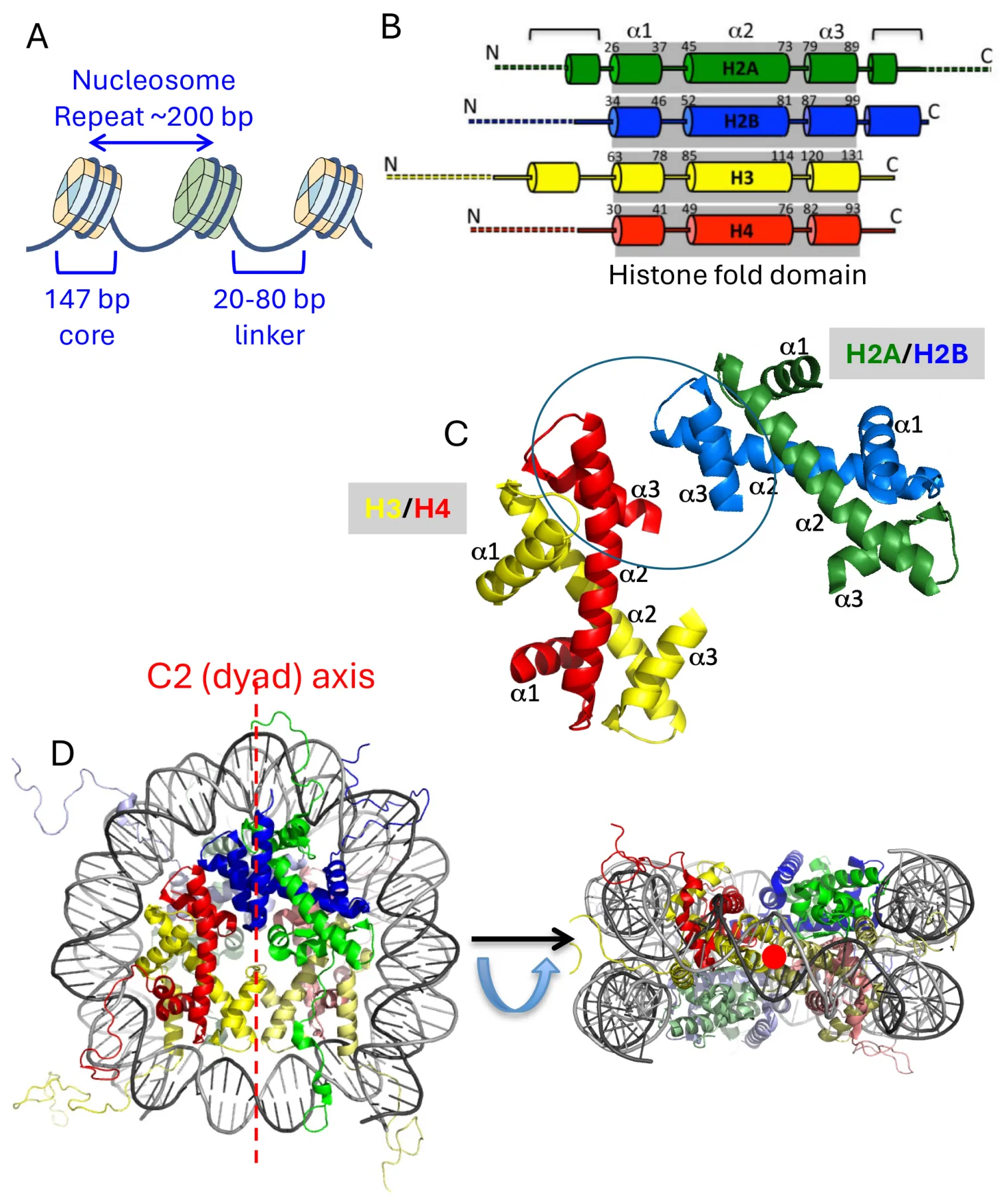
Anatomy of the nucleosome. (**A**) Three-nucleosome segment showing overall nucleosome repeat (200 bp), core DNA (147 bp) and variable linker DNA. The core histone octamer is depicted as a short column. (**B**) Linear map of the core histones showing the histone fold domain, and tail domains (dotted lines); α-helices are shown as rods. Note colors of individual histones are as indicated throughout figure. (**C**) Histone fold domains form dimerization interfaces as described in the text. A 4-helix bundle forms the dimer-dimer interface via the ends of α2 and α3 helices (oval). (**D**) Two views of the nucleosome core showing the core histones (as depicted in (**C**)) and DNA (gray). Note the dyad axis that runs through the center of the nucleosome (red dotted line). Image adapted from PDB file 1KX5 [8].

Except for small regions where they are displaced, eukaryotic DNA is saturated with nucleosomes. These “nu-bodies” or nucleosomes were first observed by Olins & Olins [1] and constitute the basic repeating structural unit of chromatin [2]. Each nucleosome unit includes a ‘nucleosome core’ containing approximately ~147 bp of nucleosome core DNA wrapped about 1.7 times around an octamer of core histones comprising two copies each of H2A, H2B, H3, and H4 [3] (Figure 1A). The remainder of the nucleosome repeat includes a variable length of linker DNA (approximately 20–90 bp), and, in most cases, a single molecule of linker histone (H1). Of note, due to the variability in linker DNA, the average nucleosome repeat in eukaryotic organisms ranges from approximately 165 bp (baker’s yeast) to 240 bp (sea urchins) [2], with most metazoans averaging ~200 bp (Figure 1A).

## 2. Anatomy of the Nucleosome

Each of the core histones has a ‘histone fold’ domain that comprises about 75% of each of these proteins [2,3] (Figure 1B). The histone fold domains are largely α-helical and constitute a large interface that mediates dimerization of H2A with H2B and H3 with H4 [4] (Figure 1C). Of note, the histone fold motif is widely distributed in nature and includes proteins that do not directly bind to DNA [5]. The four histone dimers assemble into a left-handed helical ‘spool’ onto which the core DNA is wrapped (Figure 1D). Assembly of the histone octamer occurs through end-to-end interactions among the histone dimers, with two H3/H4 dimers forming a tetramer via H3-H3 interactions, while two H2A/H2B dimers associate with either side of the (H3/H4)_2_ tetramer via H4:H2B interfaces, to form the octameric oligomeric structure [3,4,6] (Figure 1D). Notably, the octamer, as well as the nucleosome (ignoring DNA sequence identity), exhibit C_2_ symmetry about an axis referred to as the nucleosome dyad, which passes through the center of these structures (Figure 1D) [7].

The octamer formed by the histone fold domain dimers forms a left-handed spiral ramp of positive charge onto which the ~147 bp core DNA is wrapped [7]. Each dimer provides three points of interaction with the DNA, spaced about 10 bp apart, and contacting approximately 30 base pairs of DNA per dimer overall [4,6,7]. Thus, the four histone fold dimers account for about twelve contacts over ~120 bp of DNA in each nucleosome [3,7]. Of note, two additional contacts to DNA are provided via the non-histone fold N-terminal alpha-helix in H3, that seals the wrapping of the DNA at the edges of the ~147 bp nucleosome core region [3,7].

The remaining ~25–30% of the mass of each core histone is mostly contained within the N-terminal ‘tail’ domains of each histone [9]. While all four core histones contain an N-terminal ‘tail’, only H2A contains a similar C-terminal tail [2,10]. The tails are unstructured in the absence of DNA or interacting partners and project outward from the nucleosome body to contact DNA on the exterior of the nucleosome core or neighboring nucleosomes [3,8,9]. The histone tail-DNA interactions contribute only marginally to the stability of DNA wrapping in nucleosomes [11,12], but are essential for mediating inter-nucleosome interactions and the formation of condensed chromatin [13,14]. Given the role of the tail domains in mediating inter-nucleosome interactions, it is not surprising that the tails are the sites of a myriad of posttranslational epigenetic modifications that either directly or indirectly modulate these interactions to help regulate gene expression and other processes [13].

## 3. The Nucleosome Core Particle and the Chromatosome

Because of the tight association of the core DNA with the histone octamer, digestion of chromatin from virtually all eukaryotes by the endo/exonucleolytic micrococcal nuclease (MNase) produces a rather uniform entity known as the nucleosome core particle (NCP) [15,16]. The NCP contains the 147 bp core DNA and the core histone octamer, but lacks linker histones, indicating that linker DNA is required for linker histone binding to nucleosomes [17,18]. However, it is important to note that because of the sequence dependence of MNase, as well as several other factors including differential bendability of the large spectrum of DNA sequences assembled into nucleosomes in vivo, and post-translational modifications such as acetylation that weaken histone-DNA interactions, digestion of native chromatin produces NCPs with a rather wide range of sizes around an average of ~150 bp [19]. For a more complete description of the nucleosome core structure, please see Ref. [3].

More limited MNase digestion releases an intermediate product containing one molecule of full-length H1, approximately 167 bp of DNA, and the core histone octamer, termed the chromatosome by Robert Simpson in 1978 [18]. Thus, the chromatosome is a unique entity that results from micrococcal nuclease digestion of oligonucleosomes containing linker histones, and is a precursor to the nucleosome core particle [2]. The 20 bp of linker DNA within the chromatosome is evenly distributed, with approximately 10 bp on each side of the nucleosome core DNA, indicating that stable binding of H1 requires only the first 10 bp of linker DNA to either side of the core region and is indicative of ‘on-dyad’ binding behavior where the H1 is situated at the center of the nucleosome, where the dyad passes through the central wrap of DNA [17,18]. This would allow the H1 to contact each linker DNA, resulting in the protection from cleavage by MNase (see below). Of note, recent high-resolution structures of native chromatosomes have been recently obtained (also see Section 6, below) [20,21].

## 4. The Linker Histone Family

Linker histones are distinct in many ways from the core histones as they differ in structure, abundance, mobility, and contribution to chromatin. Indeed, in mammals the linker histone (H1) family is composed of a large number of subtypes. For example, in humans and mice there are seven somatic and four gametic subtypes [22]. Of the somatic subtypes, H1.1, H1.2, H1.3, H1.4, and H1.5 are expressed in a replication-dependent manner, while H1.0 and H1.10 (H1.X) are expressed throughout the cell cycle. Subtypes across species are orthologs, as they are more similar to each other than other subtypes are within each species [22]. Careful analysis via High-Performance Liquid Chromatography (HPLC) shows that subtype composition varies from tissue to tissue and during development [22,23,24,25,26]. In addition, the ratio of H1s per nucleosome increases with differentiation, which is likely due to the requirement to more generally down-regulate global transcriptional activity to match the reduced proliferative potential of the cell. In contrast, the lowest H1-nucleosome levels are observed in gametic cells, stem cells, and other pluripotent cells, where levels (ratios) are as low as 0.4 H1s per nucleosome [26]. Taken together, the maintenance of a specific set of H1 subtypes throughout biology, and the distinct combination of subtypes found in specific tissues and during development suggests that each contributes a subtly different function to the overall ensemble of “H1 activity”.

Consistent with this idea, unlike the core histones, H1s are very mobile and move rapidly about the nucleus. Fluorescence Recovery After Photobleaching (FRAP) experiments show that mouse H1.0 exhibits a 50% recovery time (t_50_) of <1 min, a time scale much faster than that typically associated with gene expression changes [27,28]. Moreover, H1s show little if any, binding specificity across genomes. Therefore, one possibility is that the ‘mix’ of H1 subtypes amounts to a precisely tuned environment for regulation of chromatin structure suitable to each cell or tissue. Moreover, while subtle differences can be found in H1 subtype distribution, all subtypes can be found rather evenly distributed throughout the entire genome [22,29]. Genomic analyses do show that H1s tend to be depleted at promoter regions, commensurate with the nucleosome-depleted region (NDR) upstream of transcription start sites of active/poised genes, or other places where nucleosomes are displaced (enhancers, insulators, replication origins) [29,30]. Interestingly, some H1 subtypes appear enriched at the nuclear periphery and co-localize with nuclear lamina-associated domains [30,31,32]. Nevertheless, the wide and relatively even distribution of all subtypes present across the genome, and their rapid exchange among binding sites suggests that unique localization of a subtype to specific genomic loci is not the primary reason why distinct subtype compositions are found in different tissues and during development.

The conservation of linker histone subtypes through evolution suggests conserved and distinct functions of these proteins. Moreover, linker histones have been shown to be essential proteins in multiple organisms. For example, although single knockouts (and multiple combinations of double knockouts) of all somatic H1 genes in mice result in no phenotypes likely due to compensation in expression by other subtypes [33,34], specific combinations of triple knockouts exhibit embryonic lethality [24]. Specifically, embryos bearing deletions of H1.2, H1.3 and H1.4, which constitute the largest fraction of H1s in most cell types, exhibit about a 50% reduction in total H1 content (to about 0.25 H1s per nucleosome) and die about d11.5 (day 11.5) [24]. Interestingly, loss of H1 content leads to a global reduction in nucleosome repeat length [24]. However, the reduction in H1 content did not lead to a genome-wide alteration in gene expression, with only about 0.6% of genes (38) exhibiting significant alteration in gene expression, with half (19) upregulated [25]. Of note, one of the genes upregulated was H1.0, likely due to an H1-content compensation mechanism. Likewise, one of the few gene expression changes in triple-H1 KO cells was found at imprinted loci, which are normally silenced in mouse ES cells, with commensurate loss of DNA and histone H3 K9 methylation that are associated with gene repression [35]. Later work in mice engineered to generate a triple KO of H1.2, H1.3, and H1.4 in B and T cells showed H1 loss resulted in increased nuclear size and chromatin decompaction in heterochromatin regions [36]. These results point to a role for linker histones in nuclear compartmentalization and heterochromatin formation.

Likewise, experiments with organisms with only a single or smaller numbers of subtypes also indicate essentiality of H1s. RNAi-based reduction in the somatic H1 transcript in Drosophila melanogaster reduced the single somatic H1 in *Drosophila melanogaster* (dH1) by ~80% and resulted in a reduction in NRL and lethality during the larval stage [37,38]. The reduction in dH1 also resulted in loss of heterochromatin, and de-repression of genes exhibiting position-effect variegation (PEV), aberrant expression of transposable elements, and loss of recruitment of the H3 K9 methylase Su(var)3-9 [37,38]. Interestingly, embryos could be rescued by expression of WT dH1 but not by dH1 lacking the globular domain or the proximal part of the C-terminal domain [39].

## 5. Linker Histone Domain Organization

**Figure 2:**
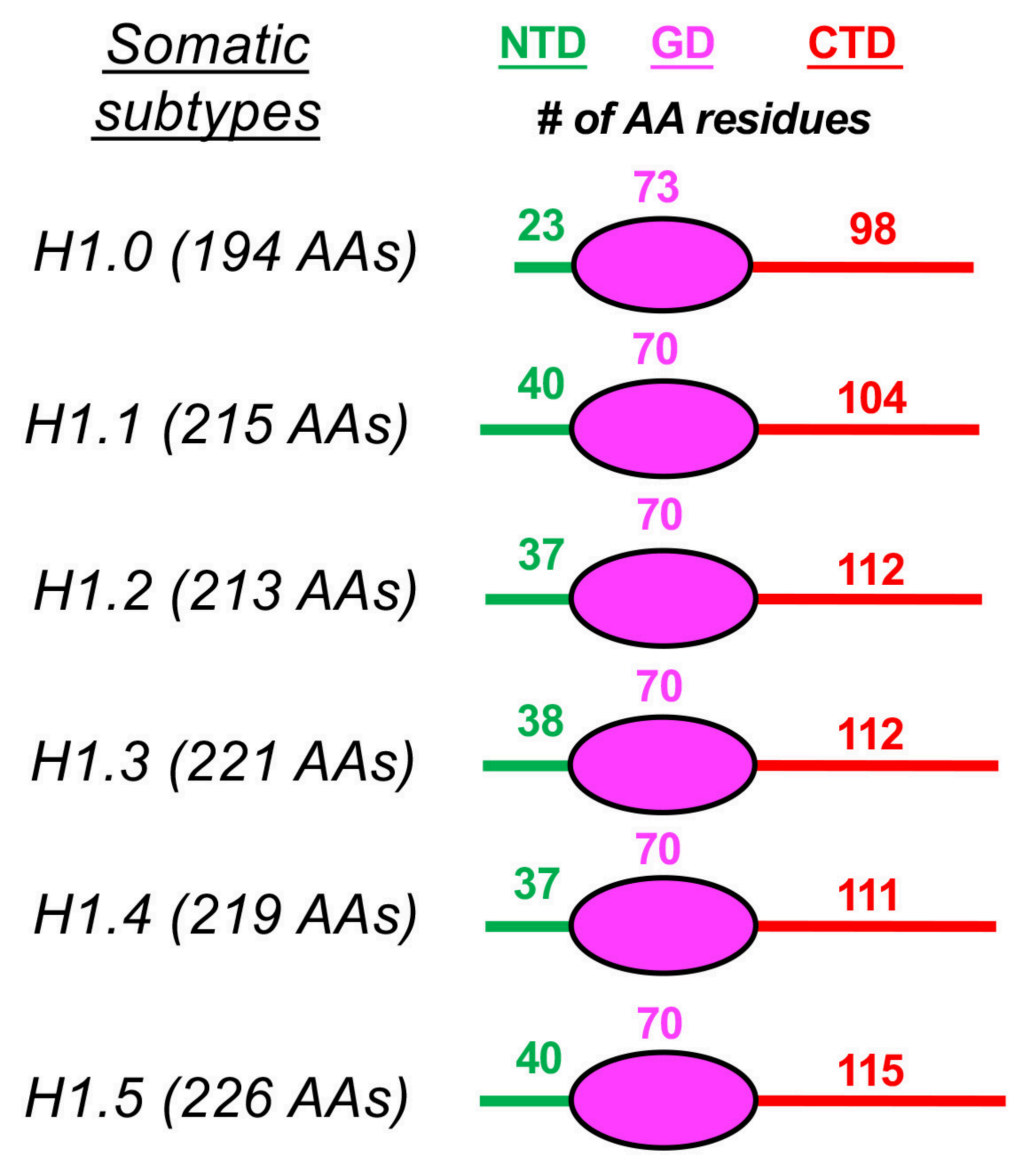
Comparison of six main human somatic H1 subtypes. The size of the N-terminal domain (NTD), the globular domain (GD) and C-terminal domain (CTD) is indicated for each protein. The residues within the NTD and CTD were determined by Alphafold prediction of residues with a pLDDT score < 85.

Although the vertebrate linker histone family comprises a wide array of subtypes, all metazoan H1s exhibit a similar tripartite structure, with a short 25–35 residue N-terminal domain (NTD), a central ~70 residue ‘globular’ domain (GD) containing a wing-helix fold, and a 100–120 residue C-terminal domain (Figure 2). The N- and C-terminal domains were originally defined by sensitivity to proteases [10] and shown by NMR and other biophysical techniques to be disordered within the free protein under physiological conditions [40], while the GD adopts a stable fold that is more resistant to proteolysis.

The NTD can be divided into two segments, an N-terminal 10–15 residue segment lacking lysines and arginines but enriched in proline, alanine, and hydrophobic residues, and therefore not expected to interact strongly with DNA, and a 5–10 residue positively charged segment with multiple lysines abutting the GD [41,42]. While data are sparse, the NTD appears to play a role in chromatin binding and may adopt a stable or meta-stable fold upon binding to nucleosomes. Biophysical approaches, including Fourier Transform Infrared Spectroscopy (FTIR) provided evidence that the NTD acquires secondary structures upon interacting with DNA or in helix-stabilizing solutions [42,43,44]. Moreover, molecular dynamics simulations of NTDs from two H1 subtypes (H1.1 and H1.2) indicate that this domain forms an amphiphilic helical structure upon interaction with nucleosomes due to charge neutralization of the lysine-rich segment, with helical propensity depending on the charge density in the basic region [45]. In addition, in vivo FRAP and in vitro studies indicate that the NTD contributes to the stability of H1 binding to chromatin [46].

**Figure 3:**
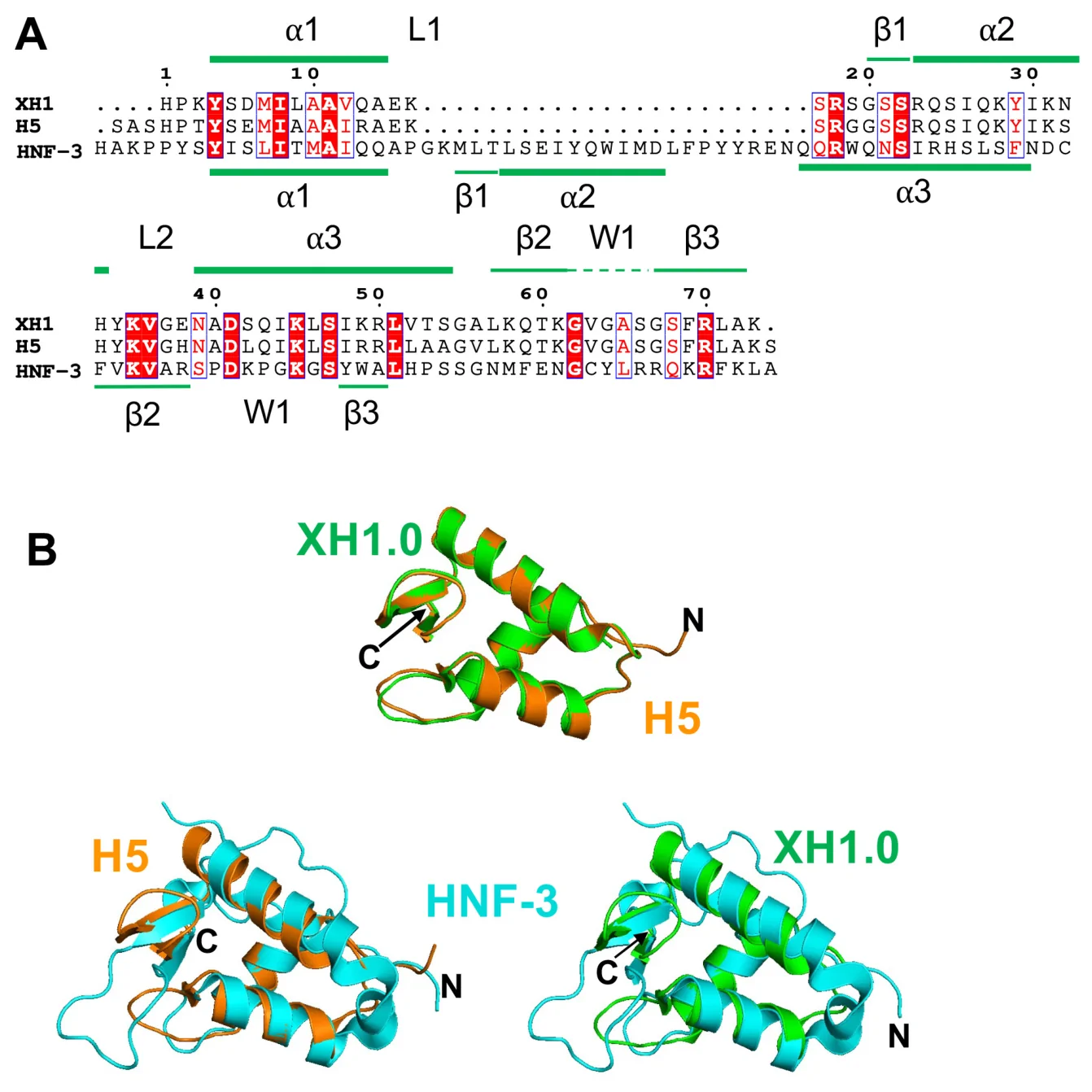
The globular domain (GD) of H1 and the winged-helix DNA binding domain of the transcription factor HNF-3 have similar structures. (**A**) Sequence alignment of the GDs from Xenopus laevis (XH1.0) and chicken H5 to the DNA binding domain of HNF-3α. Green lines above indicate secondary structures as indicated in the GDs [52]; lines below indicate the same in HNF-3 [53]. (**B**) Top—structural alignment of H5 GD to XH1 GD. Bottom left—alignment of residues 25 to 96 in H5 GD to residues 121 to 216 of HNF-3. Bottom right—alignment of XH1 GD to HNF-3. The XH1 GD, the H5 GD and HNF3 were extracted from the H1-bound nucleosome crystal structure (PDB: 5NL0), the H5-bound nucleosome crystal structure (4QLC), and the crystal structure of HNF-3 bound to DNA (PDB code: 1VTN).

The central globular domain is the primary determinant through which H1s recognize and specifically bind to the nucleosome structure. Early experiments showed that the GD, either in the context of a full-length H1 or as an isolated entity binds at the center of the nucleosome, contacting the central wrap of DNA crossing the nucleosome dyad and the two linker DNAs entering/exiting the nucleosome core [17,18]. Accordingly, the GD alone can also provide transient protection of the linker DNAs from digestion by MNase [17,47]. The GDs of several H1s were later shown by both NMR and X-ray crystallography to adopt a winged-helix fold (WHF) that is a modified version of the standard helix-turn-helix module found in many sequence-specific DNA-binding factors, with helices 2 and 3 composing the H-L-H element [48,49,50] (Figure 3). Specifically, the GD has a helix1-loop1-helix2-loop2-helix3-loop3 structure, followed by a ‘wing’ region composed of two short β-strands and a loop at the C-terminal end of the domain (Figure 3A). Of note, the GD most closely resembles the WHF of the sequence-specific DNA binding transcription factor HNF-3 (Figure 3B). Somewhat surprisingly the GD WHF recognizes and binds within the widened DNA minor groove at the dyad, while most WFH transcription factors bind via the DNA major groove [51].

**Figure 4:**
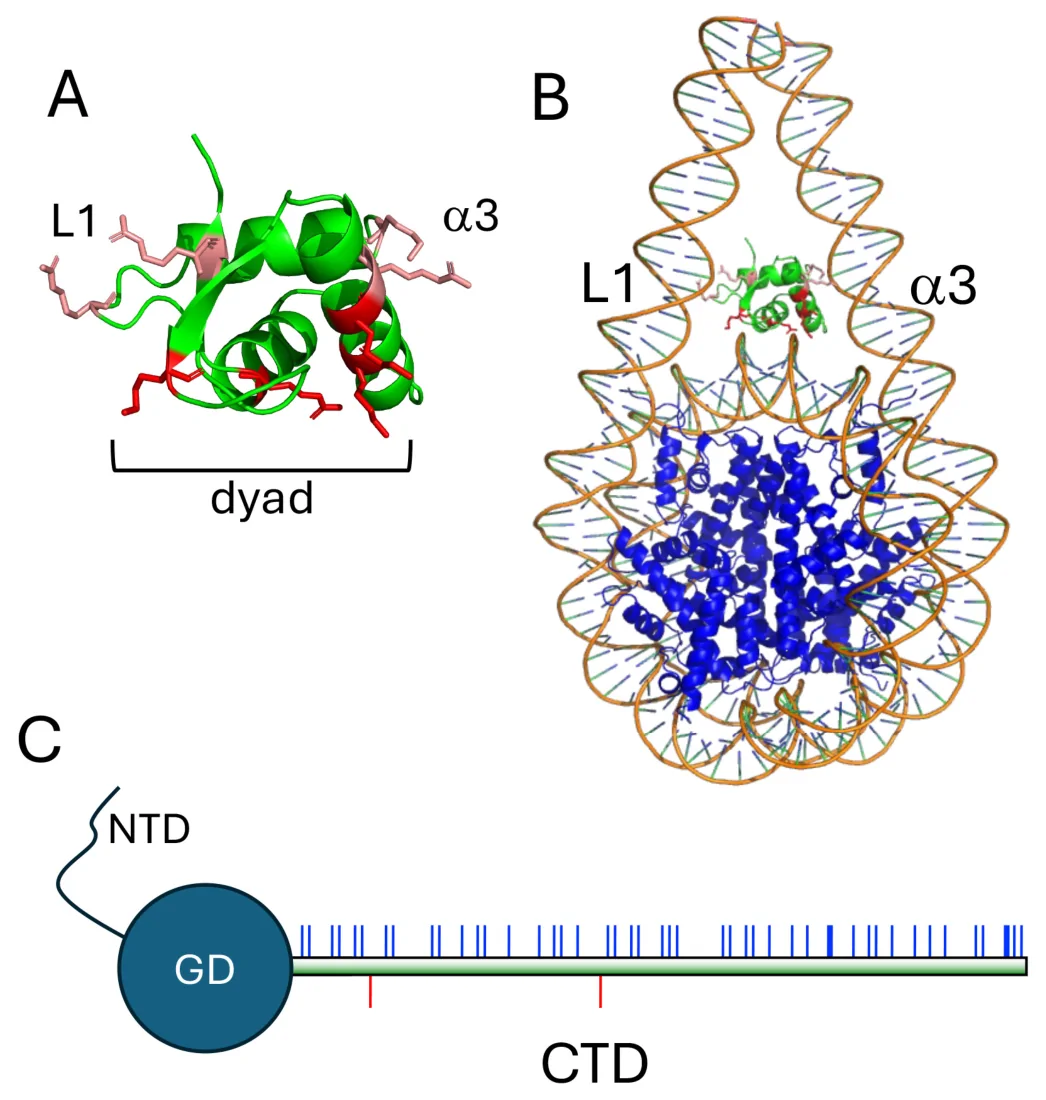
The H1 globular domain binds at the dyad position. (**A**) Cartoon model of the globular domain showing sidechains that contact the DNA at the dyad (red) and the linkers (salmon). L1 and α3 refer to loop 1 and helix 3 residues. (**B**) On-dyad binding of the H1.0 GD (green, as in (**A**)) to the nucleosome. The GD contacts the central wrap of DNA at the dyad as well as the two linker DNAs. Models based on the H1.0-nucleosome structure 5NL0 [52]. Note the NTD and the CTD are not visualized in this analysis. (**C**) Schematic showing distribution of the ~40 lysine and arginine residues (thin and thick blue lines, respectively) in the ~100 residue H1.0 CTD. The domain only has two acidic residues (red lines).

The surface of the GD is decorated with distinct patches of basic amino acid residues that interact with DNA [54,55,56], consistent with its ability to contact three DNA surfaces when bound to nucleosomes [52,57,58] (Figure 4A,B). Indeed, linker histones exhibit greater affinity for nucleosomes than for naked DNA fragments [59]. Nevertheless, in the case of the full nucleosome, crosslinking indicates that both linker DNAs are contacted by the linker histone [52]. Aside from specific recognition of the nucleosome, a major function of the GD appears to be to properly orient the CTD toward the linker DNA [52,58,60].

The highly positively charged C-terminal domain (CTD) plays an important role in neutralizing the negative charge of the DNA in condensed chromatin [50,51,52]. This ~100 residue domain typically contains about forty lysine residues distributed relatively evenly across the domain, and few if any acidic residues, depending on the H1 subtype (Figure 4C). The CTD, as mentioned above is unstructured when free in solution [40] and exhibits the sequence content emblematic of an intrinsically disordered domain [61]. However, as shown by FRET experiments, the unstructured *Xenopus* H1.0 CTD undergoes a drastic condensation upon binding to nucleosomes, both in the context of mononuclesomes and oligonucleosome arrays [62,63,64]. The extent of condensation of the CTD is influenced by the initial steps of folding of nucleosome arrays, H3 tail acetylation and phosphorylation within the CTD [19,64,65]. Interestingly the condensed state appears to include a large ensemble of various extents of condensation, that vary with chromatin condensation in a manner that suggests that the CTD interacts with the linkers of multiple nucleosomes in condensed chromatin [66,67]. However, it is clear that the H1 CTD is required for the stabilization of condensed chromatin [68].

## 6. How Do H1s Recognize and Bind to Nucleosomes?

As mentioned above, early work using MNase digestion to assess H1 binding to nucleosomes indicated that linker histones bind at the center (dyad) of the structure and protect about 10 bp of each linker DNA from digestion [18]. Moreover, it was established that the H1 GD alone provides the nucleosome structure-specific binding function for H1s [17]. However, the exact molecular details by which H1 associates with nucleosomes remained undefined until more high-resolution methods were developed. As described below, these methods confirmed that the H1 GD binds to a pocket at the dyad defined by H1 binding on the C_2_ pseudo-symmetrical axis (dyad axis) that passes through the DNA helix at the center of the nucleosome, with a binding pocket comprising three DNA surfaces: with each linker DNA, and with the central wrap of DNA in the nucleosome core, as originally envisioned by Allan and colleagues [52,57] (Figure 4B). However, some studies suggest that some H1s may bind with high probability to alternative sites on the nucleosome surface. Below, we present evidence supporting canonical ‘on-dyad’ binding as well as the evidence for ‘off-dyad’ binding modes for H1s.

## 7. Canonical On-Dyad H1 Binding Exposed!

The first high-resolution exposition of on-dyad binding by a linker histone GD was published by Zhou and colleagues [57]. These workers solved the crystal structure of a reconstituted nucleosome containing 167 bp of DNA and the GD of histone H5 (residues 23–98) at 3.5 Å resolution (PDB: 4QLC). This work showed, as expected, that the GD sits on the nucleosome dyad and contacts both ~10 bp linker DNA segments. [49,50,57]. The authors observed that the L3 loop and the N-terminal region of the α2 helix interact substantially with the central wrap of DNA crossing the dyad in the nucleosome, while the L1 loop and the α3 helix interact with each of the linker DNA strands exiting the nucleosome core [57] (Figure 4A). Thus, the convention is to refer to the L1 and α3 linker DNA strands based on these contacts.

The position of the GD within the context of a full-length H1 and a nucleosome containing complete linker DNAs was first observed in the X-ray crystallography and cryo-EM studies of Bednar and colleagues [52]. Of note, as is typical, the disordered NTD and CTD were not resolvable in the electron density maps, but the GD was observed, and its position and orientation were confirmed by site-specific crosslinking. The data showed that the GD of *Xenopus* H1.0 (an orthologue of H5) exhibited a similar, but not identical, binding orientation at the dyad site as that found by Zhou and colleagues [57]. However, the GD was shifted slightly closer to the α3 linker DNA suggesting that contacts to linker DNA by the α3 helix are more energetically favorable than contacts via the L1 loop, pulling the GD domain slightly to one side [52]. Interestingly, some additional electron density was observed along the L1 linker DNA, suggesting that the CTD was more closely aligned with the L1 rather than the α3 linker. Likewise, crosslinking data showed that only the α3 helix and not the L1 loop in the GD interacts with the sole linker in an asymmetric nucleosome containing only one linker DNA segment [52]. Consistent with this idea, prior in vivo FRAP results with H1 mutants indicated that two of the three basic sites on the surface of the GD are predominantly involved in nucleosome binding [28]. These data suggest a mechanism for orientation of the H1 GD in the event of unwrapping or unavailability of one of the two linker segments in the nucleosome [52,69]. Application of a range of other techniques, including site-specific crosslinking and hydroxyl radical footprinting, has shown that several other H1 subtypes bind the nucleosome via the canonical dyad site, including human H1.5, human H1.0, and *Xenopus* B4 [51,52].

Since this work, there have been several other high-resolution structural analyses of complete nucleosomes containing linker DNA and a full-length molecule of a linker histone. These structures all show binding of the H1 GD at approximately the center of the nucleosome. The structures of human H1.0, H1.4, and H1.10 bound to nucleosomes with 197 bp DNA were solved by cryo-EM at high resolution and showed that all three H1s bound to the nucleosome on the dyad in a similar manner [58]. However, the study showed that the three H1s differed in the extent to which they reoriented linker DNA, with H1.10 exhibiting the least reorientation. Moreover, recent high-resolution structures have been obtained confirming canonical on-dyad binding of the H1 GD in isolated native chromatosomes and in situ chromatin [70,71,72].

## 8. Alternative H1-Nucleosome Binding Modes

Despite the large number of observations showing on-dyad binding, there have been several reports of alternative H1 binding modes distinct from the canonical binding of H1 at the nucleosome dyad. An alternative binding mode was first suggested from directed cleavage, protein-DNA crosslinking and MNase studies of *Xenopus* H1.0 binding to a nucleosome containing a Xenopus 5S RNA gene [73,74,75]. These studies found that the H1 appeared to bind well off the dyad, inserted into a pocket on the inside of the superhelical gyre of DNA within the periphery of the nucleosome core region. While it is possible that these studies did not sufficiently account for alternative translational positions adopted by the nucleosome core on the 5S DNA template, no high-resolution studies of H1 bound to 5S nucleosomes have yet appeared. An off-dyad binding mode was also supported by studies combining systematic mutagenesis with in vivo FRAP determinations, which identified two main sites on the GD surface that appeared to be required for stable interaction in chromatin [28]. Modeling studies based on these results suggested that the GD binds to a site off the dyad axis, making contacts with one linker DNA and the major groove immediately adjacent to the dyad minor groove in the nucleosome [28].

**Figure 5:**
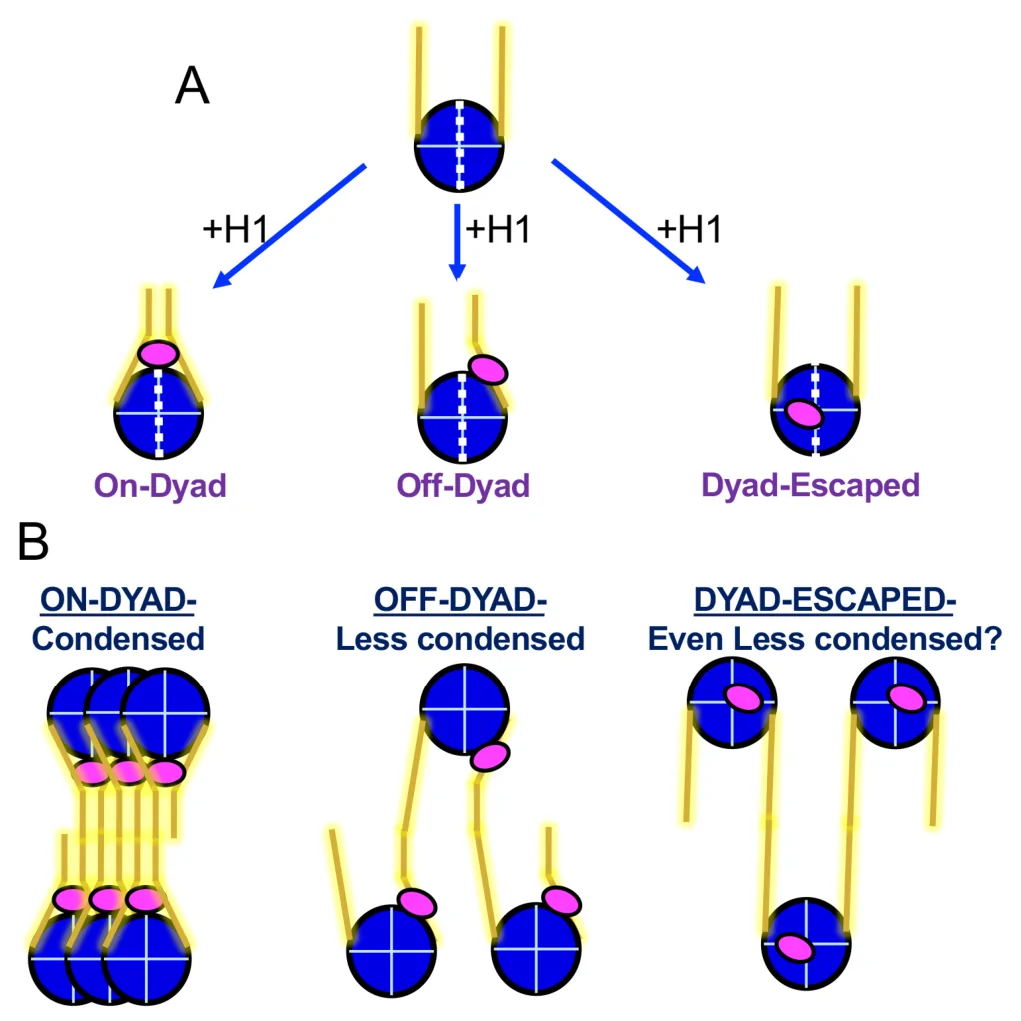
Three H1-nucleosome binding modes. (**A**) Binding modes are defined based on the position of the GD. Evidence indicates that the H1 GD may be positioned either on the dyad, off the dyad, or on the acidic patch (dyad-escaped). The canonical binding mode is taken to be on-dyad position (see text). (**B**) Distinct H1 binding-nucleosome binding modes may lead to differentially condensed chromatin structures with altered permissibility gene expression. Linker DNA is shown in gold, the core DNA in black, the core histones in blue, and the H1 GD in magenta. The dyad axis is indicated by the vertical dashed white line.

A more recent investigation of the binding location of the *Drosophila melanogaster* H1 on the nucleosome surface found that this linker histone adopted a similar off-dyad mode of binding [76] (Figure 5A). In this work, the authors performed nuclear magnetic resonance/paramagnetic relaxation enhancement (NMR/PRE) experiments, which required attaching a spin label to distinct sites within the nucleosome core that would affect the peak 1H intensities within methyl groups in close proximity to the label. This and other data indicated asymmetry in binding of the dH1 to the nucleosome, which the authors interpreted as indicating that *Drosophila* H1 (dH1) preferred to bind nucleosomes via an off-dyad mode [76]. By comparing the dH1GD sequence to that of the known on-dyad binder (chicken) H5, the authors surmised that five residues within the GD were responsible for dictating distinct nucleosome binding behavior. To test this, the five residues from the *Drosophila* H1 GD were substituted in for the analogous residues found in the H5 GD. The authors found that these substitutions switched the binding mode of chicken H5 from an on- to an off-dyad binder [77]. A potential caveat of these studies is that the dH1 protein had to be mutated at four residues within the GD to those found in the chicken H5 GD so that the protein would withstand the elevated temperatures and scan times necessary to obtain NMR spectra [76].

The off-dyad binding by select H1s poses an attractive model as it is proposed to lead to less condensed chromatin and may be emblematic of H1s that promote open chromatin and gene expression (Figure 5B). Indeed, it has been shown that binding of the dH1 GD does not alter condensation of model nucleosome arrays while binding of the H5 GD causes a modest condensation [77]. Correspondingly, H5 GD pMut, in which five residues predicted to dictate the GD binding mode have been swapped for those in dH1, also fails to condense nucleosome arrays [77]. Moreover, the GH5 binds to nucleosomes with an affinity about 4-fold greater than that of dH1 GD, while the H5 GD pMut binds similarly to dH1 [77]. Clearly, the key residue differences between H5 and dH1 GDs define differential binding affinity and affect the ability of the GD to cause modest chromatin condensation. The authors also show that the extent of chromatin condensation induced by binding the full-length H1.0 (an H5 ortholog) is slightly greater than that caused by dH1 (~37S vs. ~33S), but the experiment is done in the absence of divalent cations and at low concentrations of monovalent counterions which results in only modest levels of array compaction. Moreover, while the authors show that the GD of full-length H1.0 is bound at the dyad, they do not show that the GD of H1.0 pMut (or H5 pMut) is located off the nucleosome dyad in the context of the full-length proteins. They do, however, provide evidence that the dH1 GD is located off-dyad in the context of the full-length protein [76].

In addition to dH1, a study using cryoEM found that human H1.4 (hH1.4) also bound in an ‘off-dyad’ manner within a 12-nucleosome array. [78]. However, this structure was solved at a very low resolution (~11 Å), likely too low to unambiguously map the position of the H1 GD on the nucleosome [78]. Moreover, a recent study found that H1.4 in a 4-nucleosome array occupies the canonical on-dyad mode for several linker DNA lengths [79] and binds in the on-dyad mode in the context of a 197 bp mononuclesome in a 2.76 Å cryo-EM structure [58]. Additionally, these authors found that the glutaraldehyde crosslinking used in the original hH1.4/12-nucleosome array study likely induced off-dyad binding due to chemical modification of the H1.4 GD, which altered its ability to bind to the dyad site [80]. Moreover, chicken H5 was also observed to bind off-dyad in the context of a 12-mer array in the presence of glutaraldehyde crosslinking via a high-resolution (3.6 Å) cryo-EM structure [81]. Interestingly, in this report, the authors observed avian H5 to bind via a 3-contact, and not 2-contact, off-dyad binding mode, in which one contact came from the CTD, in contrast to the 3.5 Å crystal structure of avian H5 GD bound to a mononucleosome [57]. Thus, H1 GDs appear to be particularly susceptible to modification by glutaraldehyde, which alters their ability to exhibit native binding [80,81].

## 9. A Special Case? The “Dyad-Escaped” Binding Mode

Most recently, a third mode of H1-nucleosome binding has been proposed, in which H1 presumably loosely associates with the nucleosome acidic patch, and has been referred to as the ‘on-head’ or ‘dyad-escaped’ mode of binding [82] (Figure 5A). The ‘dyad escaped’ mode of binding was predicted by computational modeling [83] for the *Xenopus laevis* H1.0 subtype and proposed in a recent report based on atomic force microscopy (AFM) studies of human H1.5 bound to nucleosomes that were enriched for the centromeric H3 variant CENP-A [82]. The authors observed that the nucleosomes had an unusual height dimension in such studies, suggesting that the bulk of the mass of the H1.5 was located on the nucleosome protein surface, presumably interacting with the acidic patch [82]. It is predicted that the dyad-escaped mode would potentially be correlated with a state of high transcriptional permissibility (Figure 5B).

In summary, based on the structural information available to date, the nucleosome-recognizing H1 globular (GH1) domain of various H1s has been reported to bind to the nucleosome in multiple fashions as defined by where the H1 globular (G) domain is located in the nucleosome: on-dyad; 16 reports, off-dyad; five reports, and very recently a dyad-escaped or on-head mode of binding (one report) [21,52,57,58,71,72,76,77,78,79,81,82,84,85]. The reported on-dyad binding mode is defined by H1 binding on the C_2_ pseudo-symmetrical axis (dyad axis) that passes through the center of the nucleosome, in a binding pocket, in which H1 makes three DNA surface contacts (one with each linker DNA and one with the central wrap of DNA, or core DNA, in the nucleosome that passes through the dyad axis) [52,77]. Importantly, even the on-dyad mode is not explicitly uniform as there are differences in the orientation and placement of the GD that appear to depend on sequence (see [84]). While the reported off-dyad binding mode is defined by H1 binding askew from or off-center of the dyad axis that passes through the center of the nucleosome, it makes only two DNA surface contacts (one with a linker DNA and one with the central wrap of DNA in the nucleosome) [76,77]. Interestingly, the difference between the on- and off-dyad binding modes has been attributed to five key residues in the G domain [77]. Finally, it has been proposed that H1 can also populate a state in the nucleosome where it is loosely associated with the acidic patch (‘on-head’ or dyad-escaped mode of binding). All three reported binding modes are shown in Figure 5.

## 10. Even More Ways H1 Binds in Chromatin

It has been known for decades that H1s preferentially bind to DNA crossovers and other closely apposed DNA helices with high affinities [86,87,88,89]. It is not surprising then that such sites might be significantly populated in chromatin, especially in cells where the ratio of H1 to nucleosomes is >1 [26]. Indeed, nuclei of quiescent cells have much more than 1 H1 per nucleosome [23], and nucleosomes have been isolated from cells containing more than one bound H1 [90]. To address the question of where such H1s might reside in native chromatin, the Davey group has determined the structures of ‘cohesive end’ dinucleosomes, in which the linker DNAs on adjacent disomes can anneal via a complementary sequence to generate a continuous oligonucleosome strand that, in the crystal environment, approximates the nucleosome packing found in condensed chromatin. Interestingly, while the authors observe that H1 binds via the classical on-dyad mode, they also observe a second “non-dyad” mode of H1 binding in which the H1 GD binds contacts three DNA surfaces in close brought in apposition from neighboring nucleosomes [91]. Interestingly, the ‘non-dyad’ binding H1s appear to prefer interacting with highly curved DNA, perhaps because of the need for the GD to bind in a widened minor groove [91]. Taken together, these reports further elucidate alternative H1 binding modes when more than 1 H1 is present per nucleosome, with the additional non-dyad binding serving to further stabilize condensed chromatin, consistent with the repressive chromatin environment found in such cells. An additional study from this same group showed that linker DNA from adjacent nucleosomes can also define a “non-dyad” binding site. However, most if not all variations in the “non-dyad” mode of binding appear to involve at least one DNA helix wrapped within a nucleosome core, supporting the idea that H1 GDs prefer open minor grooves in highly bent DNA. These authors propose a model where H1 first occupies the canonical dyad site with additional H1s seeking “non-dyad” sites with similar features that serve to stabilize the condensed chromatin [92]. In addition, while these authors have shown that the specific characteristics of the novel non-dyad H1 binding appear to be dependent on defined linker length variation in their model chromatin complexes [91,92], it remains unclear whether the highly heterogeneous linker DNA lengths found in native chromatin affect H1 interactions.

## 11. Conclusions and Future Objectives

While the basic structures and functions of the core histones are fairly well understood, we may be reaching the tip of the iceberg in regard to our structural understanding of how H1 associates with chromatin. H1s bind DNA with affinities only 2-4-fold less than their association with the canonical on-dyad position in nucleosomes. Given the rapidity with which H1s exchange between binding sites in nuclei, coupled with the high concentration of DNA surfaces, it might be expected that ‘non-dyad’ binding occurs with high frequency. Current evidence indicates that some of this binding occurs at positions that resemble the on-dyad binding pocket, where the H1 bridges sites of bent DNA wrapped around the nucleosome core and additional DNA surfaces in close proximity. In addition, DNA in the nucleosome is known to spontaneously unwrap from the periphery of the nucleosome core, leaving only two of the three DNA surfaces in close juxtaposition. Nevertheless, crosslinking data indicates that H1 remains bound, with a specific orientation involving the α3 helix remaining in association with the α3 linker. Some H1 subtypes may prefer binding to off-dyad sites slightly shifted from the on-dyad position or even at locations in which the GD does not interact with DNA at all. Further investigation is certainly warranted to confirm whether these different H1-nucleosome binding modes exist in vivo and, if so, how they affect chromatin structure. 

Many important questions remain. For example, what are the distinct contributions provided by the seemingly very similar somatic H1 subtypes found in the nuclei of higher organisms, and why does the proportion of each subtype vary among tissues? Do distinct H1 subtypes have specific functions amongst H1s in regulating chromatin structure and function? What is the distribution of H1 among the various binding modes in vivo, and how does the distribution between the canonical on-dyad binding mode and alternative modes in native chromatin depend on the total H1 complements found in different cell types? Finally, little is known regarding interactions of the NTD and especially the CTD in nucleosomes and chromatin, and how these interactions might depend on the precise location of the GD. The CTD has been shown to be required for H1-dependent chromatin condensation and is an essential domain in *Drosophila*.

## Data Availability

No new data were created or analyzed in this study. Data sharing is not applicable to this article.

## Abbreviations

The following abbreviations are used in this manuscript:

WHF	Winged helix fold
GD	Globular domain
CD	Circular dichroism
CTD	C-terminal domain
NTD	N-terminal domain
MNase	Micrococcal nuclease
AFM	Atomic Force Microscopy
FRAP	Fluorescence recovery after photobleaching
FRET	Förster resonance energy transfer
cryoEM	Cryo-electron microscopy
FTIR	Fourier transform infrared spectroscopy
